# Integrating bulk and single‐cell data to predict the prognosis and identify the immune landscape in HNSCC

**DOI:** 10.1111/jcmm.18009

**Published:** 2023-10-26

**Authors:** Chunlong Yang, Xiaoning Cheng, Shenglan Gao, Qingjun Pan

**Affiliations:** ^1^ Clinical Research Center Affiliated Hospital of Guangdong Medical University Zhanjiang China; ^2^ Zhanjiang Central Hospital Guangdong Medical University Zhanjiang China

**Keywords:** endothelial cell, HNSCC, risk model, Scissor, single‐cell data

## Abstract

The complex interplay between tumour cells and the tumour microenvironment (TME) underscores the necessity for gaining comprehensive insights into disease progression. This study centres on elucidating the elusive the elusive role of endothelial cells within the TME of head and neck squamous cell carcinoma (HNSCC). Despite their crucial involvement in angiogenesis and vascular function, the mechanistic diversity of endothelial cells among HNSCC patients remains largely uncharted. Leveraging advanced single‐cell RNA sequencing (scRNA‐Seq) technology and the Scissor algorithm, we aimed to bridge this knowledge gap and illuminate the intricate interplay between endothelial cells and patient prognosis within the context of HNSCC. Here, endothelial cells were categorized into Scissor^high^ and Scissor^low^ subtypes. We identified Scissor^+^ endothelial cells exhibiting pro‐tumorigenic profiles and constructed a prognostic risk model for HNSCC. Additionally, four biomarkers also were identified by analysing the gene expression profiles of patients with HNSCC and a prognostic risk prediction model was constructed based on these genes. Furthermore, the correlations between endothelial cells and prognosis of patients with HNSCC were analysed by integrating bulk and single‐cell sequencing data, revealing a close association between SHSS and the overall survival (OS) of HNSCC patients with malignant endothelial cells. Finally, we validated the prognostic model by RT‐qPCR and IHC analysis. These findings enhance our comprehension of TME heterogeneity at the single‐cell level and provide a prognostic model for HNSCC.

## 1 BACKGROUND

Cancer is a highly heterogeneous disease that involves frequent interactions between tumour cells and the tumour microenvironment (TME). TME consists of various immune and mesenchymal stromal‐derived cells as well as the extracellular matrix, which interact directly or indirectly with malignant cells and influence tumour progression.^1^, ^2^, ^3^ TME components function as a double‐edged sword by either promoting or inhibiting tumour progression. As important stromal cells in the TME, endothelial cells play a key role in the formation and function of blood and lymphatic vessels. Excessive proliferation and abnormalities in endothelial cells may lead to angiogenesis, lymph angiogenesis, or abnormal vascular function, contributing to tumour development.^4^, ^5^, ^6^ Under the influence of some pro‐angiogenic factors, endothelial cells form a barrier to inhibit T‐cell infiltration into the TME.^7^ Tumour cells induce endothelial cells to convert to a fetal‐like phenotype by secreting the vascular endothelial growth factor, which affects tumour‐associated macrophages via the Notch pathway, shaping the immunosuppressive microenvironment.^8^ Over the past few decades, significant breakthroughs in tumour therapy targeting tumour angiogenesis have been achieved.^9^, ^10^, ^11^ It is necessary to explore the roles of endothelial cells in tumours and determine their association with patient prognosis to develop new targets for the treatment of tumours.

Head and neck cancer is a leading cause of tumour‐related deaths, accounting for approximately 300,000 annual deaths worldwide.^12^ Head and neck squamous cell carcinomas (HNSCC) are the most common histological subtype, accounting for approximately 95% of head and neck tumours. It is highly aggressive and heterogeneous, with poor prognostic outcome. Studies have reported the presence of various cell types, including immune cells, endothelial cells and fibroblasts, in the HNSCC TME.^13^, ^14^ Therefore, characteristic parameters of the TME in HNSCC are important discriminatory factors for the clinical staging and survival prognosis of patients. For instance, the abundance of tumour‐infiltrating lymphocytes has been reported to be correlated to the prognosis of patients with HNSCC.^15^, ^16^ Within the intricate milieu of the TMEs, endothelial cells emerge as a pivotal stromal component orchestrating angiogenesis and vascular function.^17^ However, the exact mechanisms by which endothelial cells manifest their diversity among HNSCC patients remain elusive, representing a notable gap in our understanding. This knowledge gap is of paramount importance, as it holds the key to deciphering the intricate TME dynamics and their impact on disease progression and patient outcomes.

Single‐cell RNA sequencing (scRNA‐seq) technology has greatly improved our understanding of tumour pathogenesis and TME composition.^17^, ^18^ scRNA‐Seq analysis can identify specific immune cell subsets for the development of new therapies against HNSCC. Notably, the recently developed Scissor algorithm can identify highly phenotypically relevant cell subpopulations from scRNA‐Seq data based on bulk RNA‐Seq phenotypes.^19^, ^20^ In this study, we obtained HNSCC scRNA‐Seq data from the Gene Expression Omnibus (GEO) database and used the Scissor algorithm to extract the subpopulations of endothelial cells associated with poor patient prognosis. Combined with The Cancer Genome Atlas (TCGA) transcriptomic data, we identified four biomarkers closely associated with patient prognosis. We also constructed a prognostic risk prediction model based on these four genes using the Cox proportional hazards model and validated it in a different HNSCC patient cohort.

## 2 METHODS

### 2.1 Data download

Single‐cell transcriptomes and transcriptomic and clinical information of HNSCC samples were downloaded from TCGA (https://www.cancer.gov/tcga) and GEO (http://www.ncbi.nlm.nih.gov/geo/) databases. scRNA‐Seq data for HNSCC were obtained from the GSE164690 dataset for further analysis of endothelial cell clusters. This dataset was chosen for its comprehensive representation of endothelial cell clusters, crucial for our study's objectives. By utilizing data from a diverse range of samples, we aimed to minimize potential bias and ensure that our findings generalize across patient populations. Transcriptome expression data, genomic mutation data and corresponding clinical annotations of HNSCC were obtained from TCGA database. Additionally, transcriptome expression data from the GSE65858 dataset were included for independent validation purposes, demonstrating the reliability and reproducibility of our prognostic model.

### 2.2 scRNA‐Seq data processing and cell clustering

Seurat (version 4.1.1) package of the R software was used to analyse the scRNA‐seq data of HNSCC samples.^21^ The ‘harmony’ function was used to eliminate the batch effects before further analysis. Briefly, we first normalized the gene expression matrix and then used ‘FindVariableFeatures’ to identify 2000 highly variable genes. The latter was used to perform principal component analysis to reduce the dimensionality of the data. Moreover, ‘FindNeighbors’ and ‘FindClusters’ functions were used to cluster and identify the cells. Cell clusters were then visualized on a two‐dimensional plot using the ‘RunUMAP’ function. Marker genes in each cluster were obtained using the ‘FindAllMarkers’ function. Besides, to identify tumour cells within the epithelial population, we employed the ‘copyKAT’ algorithm to assess the copy number variations (CNVs) of single cells, enabling the discrimination of tumour cells based on their distinctive genomic profiles. By analysing the CNVs of individual cells, we were able to accurately pinpoint the presence of tumour cells within the epithelial population.

### 2.3 SCISSOR analysis and identification of Scissor high score signature (SHSS)

SCISSOR (version 2.0.0) was used to integrate the GSE164690 scRNA‐seq data with phenotypically correlated TCGA bulk RNA‐Seq data. Endothelial cell populations were first determined to correlate with bulk samples, and their regression models were then optimized to correlate with sample phenotypes using the SCISSOR function to identify different phenotypic subpopulations of endothelial cells. Specifically, it employs linear regression for continuous variables, logistic regression for dichotomous variables, and Cox regression for clinical survival data. Aiming to capture the most influential cells shaping the phenotype of interest, SCISSOR incorporates a sparse penalty and graph regularization into the regression model. As a result of this meticulous process, SCISSOR‐positive (Scissor^+^) cells and SCISSOR‐negative (Scissor^−^) cells are discerned based on the signs of the estimated regression coefficients. Scissor^+^ cells are positively associated with the phenotype of interest, while Scissor^−^ cells exhibit a negative association. Cells with coefficients of zero are designated as background cells, aiding in the robust identification of subpopulations. After obtaining SCISSOR‐classified endothelial cell subpopulations, we defined the differentially expressed genes (DEGs) that were highly expressed by Scissor‐positive cells (p < 0.05, |log2FC| > 0.25) as the SHSS and used them for subsequent classification analysis.

### 2.4 Cell–cell communication analysis

We used the ‘CellChat’ (version 1.5.0) R package to infer cell–cell communication between the six‐cell clusters. We divided single‐cell samples of HNSCC into two groups according to SHSS and compared discrepancies in ligand and receptor interactions and signalling pathways between the two groups.

### 2.5 Gene mutation analysis

As previously described, we downloaded copy number variation (CNV) data of HNSCC patients from TCGA database. We then analysed and visualized the mutation levels between the two groups using the ‘map tool’ package.

### 2.6 GO enrichment analysis

To define the molecular biological functions of the two groups classified by SHSS, we obtained differentially expressed genes (| log2FC | > 1 and *p* < 0.05) between the two groups using the ‘limma’ package. To investigate the enriched signalling pathways in the two groups, we performed gene ontology (GO) biological processes analysis using the ‘clusterProfiler’ package, and the GO term with *p* < 0.05 was statistically significant.

### 2.7 Immune infiltration analysis and determination of the tumour immune dysfunction and exclusion (TIDE) score

According to the TCGA and GSE65858 HNSCC transcriptome data, the single‐sample gene set enrichment analysis (ssGSEA) was performed to calculate the infiltration score of 28 immune‐related cells in the TME between the two groups. CIBERSORT was used to calculate the proportion of the major 22 immune cell subpopulations between the two groups. To assess the tumour immune evasion ability, TIDE scores were calculated between the groups using online software (http://tide.dfci.harvard.edu).

### 2.8 Construction and validation of the SHSS prognostic risk model

TCGA and GSE65858 HNSCC cases were included to establish a prognostic risk model. Briefly, to screen the genes most associated with prognosis from the SHSS, we performed the least absolute shrinkage and selection operator (LASSO) regression using the ‘glmnet’ (version 4.1.4) R package. Forest plots were obtained by univariate and bivariate Cox regression analysis using the ‘forest plot’ package in R. Genes with hazard ratio (HR) > 1 were considered risk factors and HR <1 were protective factors. The risk score for each patient was calculated as follows: risk score = h0*e^∑i = 0nexp(). Patients were stratified according to the risk score, and stratified survival curves were plotted using the Kaplan–Meier method. The accuracy of the prognostic model was evaluated using a time‐dependent receiver operating characteristic curve. We then conducted uni‐cox and multi‐cox analyses to investigate the relationship between risk scores, clinicopathological characteristics, and overall survival (OS) in HNSCC. We generated a nomogram using the ‘rms’ R package (version 6.3.0) to predict the 1‐, 2‐ and 3‐year OS of HNSCC. Finally, we developed calibration curves to assess the accuracy of nomogram‐predicted OS.

### 2.9 Patient tissue samples

HNSCC tumour tissues were collected from 20 patients who underwent surgical ectomy at the Department of Oral and Maxillofacial Surgery, First Affiliated Hospital, Sun Yat‐Sen University. Informed consents were obtained from all patients prior to analysis. Clinical characteristics of patients were summarized in Table S1. All patient‐related studies were reviewed and approved by the Institutional Review Board of the First Affiliated Hospital of Sun Yat‐Sen University. The original data of RT‐qPCR and IHC‐score were also deposited in Table S1.

### 2.10 IHC staining

The tissue samples were fixed in 10% buffered formalin at room temperature overnight and subsequently transferred to 70% ethanol for preservation. The samples were then embedded in liquid paraffin and subjected to two consecutive 10‐hour permeabilization treatments, followed by an overnight heating at 60°C. The prepared paraffin blocks were sectioned into 5 μm slices, which were then dried and deparaffinized in 4°C ethanol. The sections were subsequently rehydrated in phosphate‐buffered saline (PBS) containing 1% bovine serum albumin (BSA) to block nonspecific binding sites. After an overnight incubation with the appropriate primary antibody at an optimal concentration, the sections were incubated with PBS and HRP‐secondary antibody at an appropriate concentration for 30 minutes. Finally, the sections were stained with DAB staining solution in PBS and counterstained with neutral background reagent. Microscopic observations were made, and image analysis software was used to analyse the sections. The primary antibodies used in this study included CCND1 (SAB, 31064, 1:200), FTH1 (CST, 3998S, 1:200), YWHAG (Abcam, ab248822, 1:200) and TNFRSF12A (Abnova, MAB21331, 1:200).

### 2.11 Real‐time quantitative PCR

Total RNA was isolated from tissue samples of head and neck squamous cell carcinoma using the TRIzol reagent (ThermoFisher, 15596018). The concentration and purity of the RNA were evaluated using a NanoDrop 2000 spectrophotometer. Subsequently, cDNA was synthesized from the RNA using the Vazyme Reverse Transcription kit (R312‐01), following the manufacturer's protocol. Real‐time PCR was performed on 1 μg of cDNA using the SYBR Green™ Master Mix (AG, AG11701). Technical replicates were conducted at least three times for each sample to ensure reproducibility. *GAPDH* was used as a reference gene to quantify the target mRNA. The primer pairs used for Real‐time PCR amplification were as follows: *CCND1* (forward: 5′‐ TGGAGCCCGTGAAAAAGAGC‐3′; reverse: 5′‐TCTCCTTCATCTTAGAGGCCAC‐3′), *FTH1* (forward: 5′‐ CCCCATTTGTGTGACTTCAT −3′; reverse: 5′‐GCCCGAGGCTTAGCTTTCATT‐3′), *YWHAG* (forward: 5′‐ AGCCACTGTCGAATGAGGAAC‐3′; reverse: 5′‐ CTGCTCAATGCTACTGATGACC‐3′), *TNFRSF12A* (forward: 5′‐ AAATCATTCGGGAGGAGGTGGGA‐3′; reverse: 5′‐ CAGCTGTTTTGTGTGAGCCAGC‐3′) and *GAPDH* (forward: 5′‐ AGATCCCTCCAAAATCAAGTGG‐3′; reverse: 5′‐ GGCAGAGATGATGACCCTTTT −3′).

### 2.12 Statistical analyses

All analyses were conducted using the R software (version 4.0.0; http://www.R‐project.org). Log‐rank test was used to calculate the *p*‐value for overall survival. Univariate and multivariate Cox regression analyses were used to evaluate the independent prognostic value of all clinical factors. Statistical significance was set at *p* < 0.05.

## 3 RESULTS

### 3.1 Identification of endothelial cell subclusters associated with poor prognosis

**FIGURE 1:**
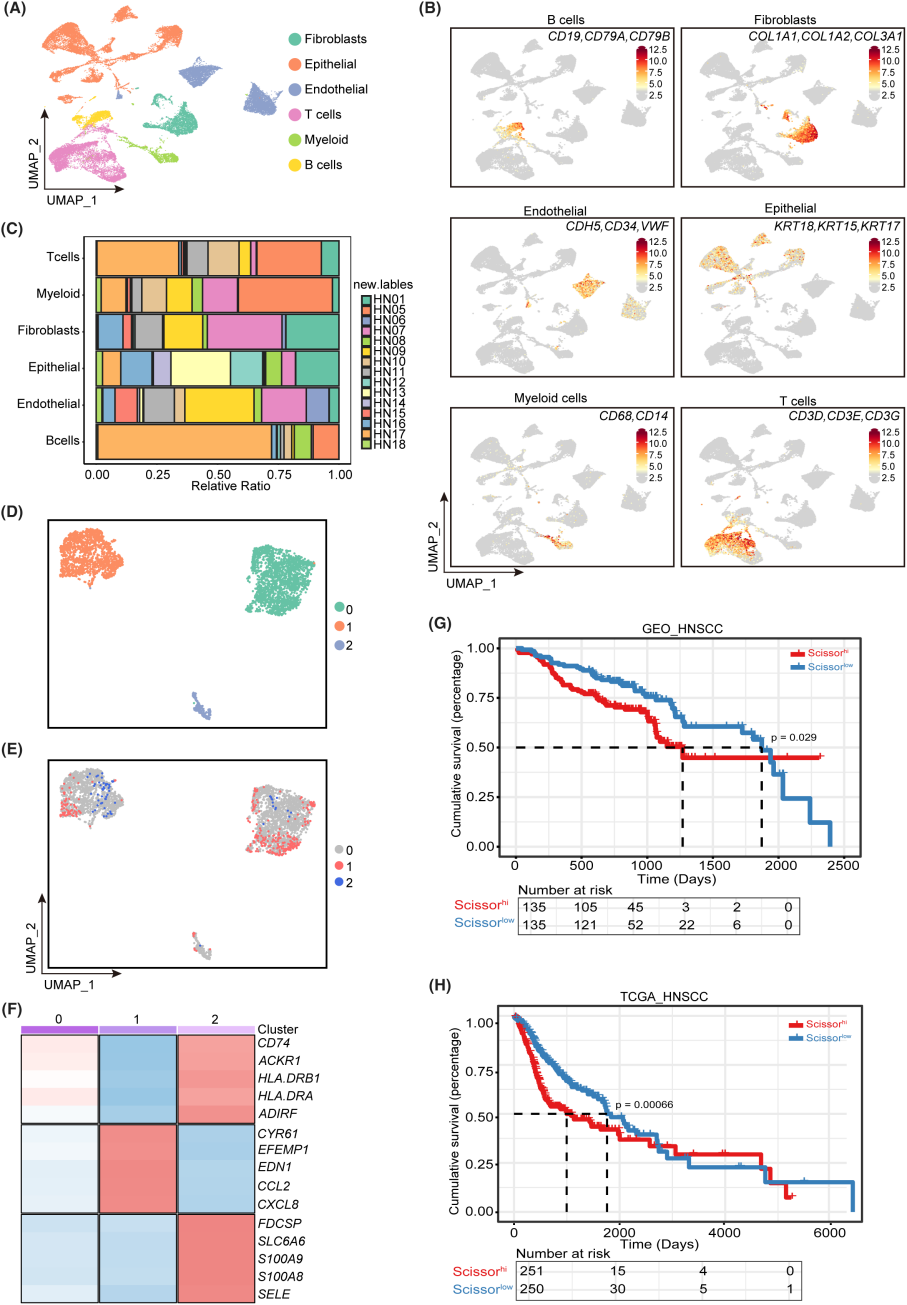
Identification of endothelial characteristic gene sets associated with poor prognosis via single‐cell analysis. (A) Uniform manifold approximation and projection (UMAP) plot showing a projection of single‐cell RNA sequencing (scRNA‐Seq) cluster labels on cells from 15 samples in the head and neck squamous cell carcinoma (HNSCC) scRNA‐seq dataset. Subpopulations of epithelial cells (Coral), B cells (Yellow), endothelial cells (LightSteelBlue), fibroblasts (Aquamarine), T cells (Pink) and myeloid cells (LawnGreen) were identified and each cell type was coloured. (B) UMAP plot showing the expression levels of characteristic gene sets in all cell types. (C) Bar graph showing the proportion of cell types in each HNSCC sample. (D) UMAP plot showing three tumour‐associated endothelial cell clusters. (E) UMAP plot showing three clusters of tumour‐associated endothelial cells differentiated using Scissors. Cluster 1 represents the malignant endothelial cells. (F) Heatmap of the signature genes in different tumour‐associated endothelial cell clusters. Each cell cluster was represented by five specifically expressed genes. (G) Kaplan–Meier survival curve illustrating the clinical relevance of the Scissor signature in the HNSCC Gene Expression Omnibus (GEO) dataset. (H) Kaplan–Meier survival curve illustrating the clinical relevance of the Scissor signature in the HNSCC The Cancer Genome Atlas (TCGA) dataset.

To evaluate TME cell composition and cellular status in HNSCC, we downloaded single‐cell sequencing data from 15 samples from the GEO database (GSE164690). After data quality control, we obtained 34,464 cells. We identified distinct fibroblasts, epithelial cells, endothelial cells, T cells, myeloid cells and B cells using characteristic genes defined by typical cell types (Figure 1A,B). The heterogeneity of tumours was also demonstrated by the different proportions of various cell types in the different samples (Figure 1C).

To further explore the characteristics of endothelial cells in HNSCC, we performed unbiased secondary clustering of endothelial cells and identified three endothelial sub‐clusters (Figure 1D). We applied scissor analysis to HNSCC scRNA‐Seq data to determine the association between endothelial cells and patient prognosis using 520 TCGA‐HNSCC bulk samples to guide phenotype analysis. Among the 3664 endothelial cells, 316 Scissor^+^ endothelial cells (cluster 1), 62 Scissor^−^ endothelial cells (cluster 2) and 3286 background cells (cluster 0) were obtained (Figure 1E), and Cluster 1 was associated with poor patient prognosis. We further identified DEGs in the endothelial cell subpopulations based on the Scissor subpopulation and found that the DEGs of Cluster 1 (*CCL2* and *CXCL8*) exhibited a pro‐tumorigenic profile, whereas those of Cluster 2 (*S100A8/A9* and *CD74*) exhibited pro‐inflammatory and anti‐tumorigenic features (Figure 1F). We then defined the 492 upregulated DEGs of Cluster 1 as the SHSS and applied them to TCGA and GEO HNSCC patient cohort stratification (METHOD). We found that patients with higher signature scores (Scissor^high^) had significantly worse survival rates than those with lower signature scores (Scissor^low^) in both cohorts (Figure 1G,H).

### 3.2 Characterization of the HNSCC TME based on SHSS classification

**FIGURE 2:**
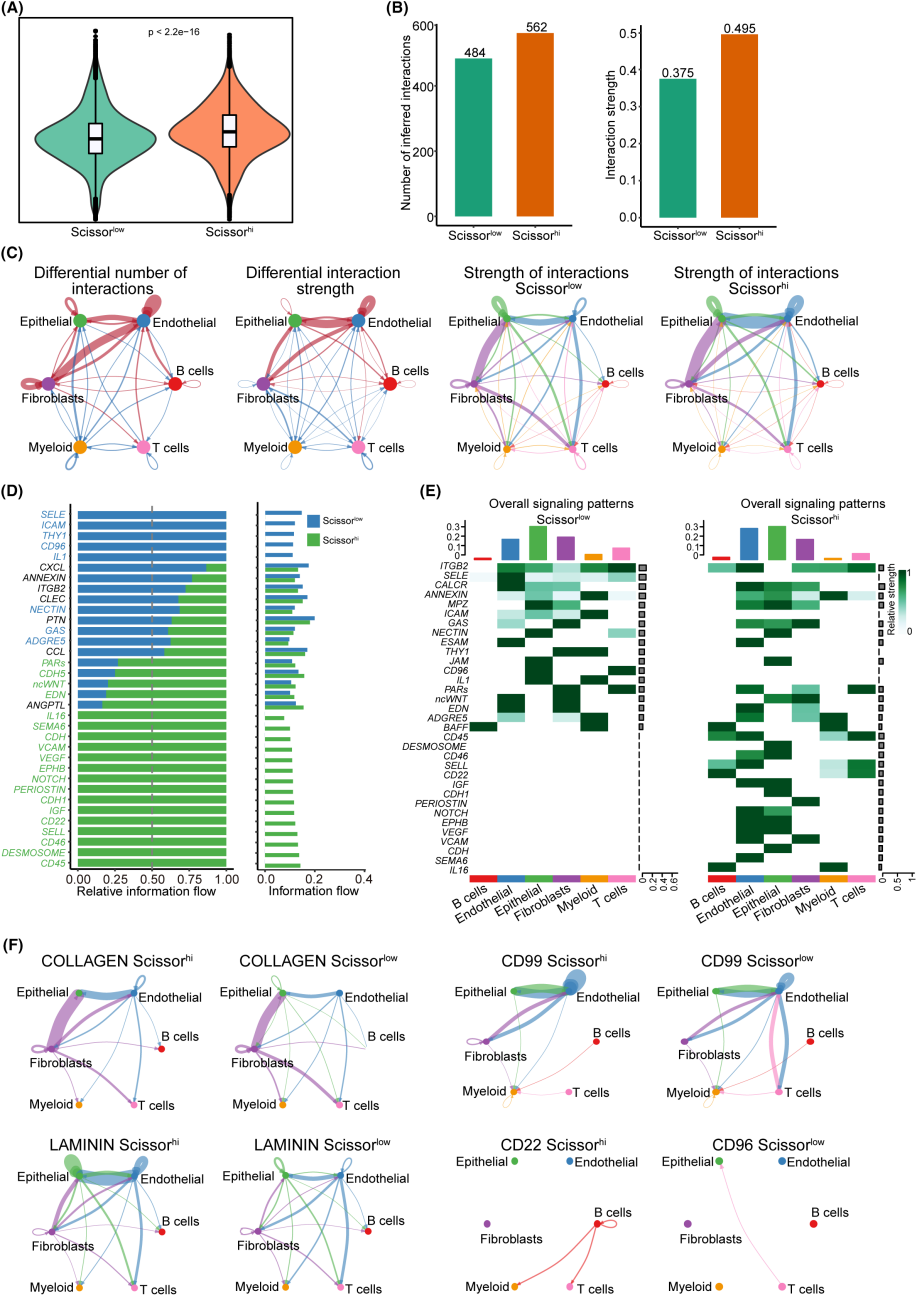
Molecular characteristics and cell–cell communications in Scissor^low^ and Scissor^high^ clusters. (A) Violin plot showing the signature scores of Scissor^high^ genes in the two clusters of HNSCC scRNA‐seq data. p‐value was determined using a two‐tailed unpaired Student's *t*‐test. (B) Histogram showing the differential number of interactions (left) or interaction strength (right) in the cell–cell communication network between the two groups. (C) Network plot showing the difference in the capacity for intercellular communication between the two groups. Blue line indicates the downregulation of intercellular communication, red line indicates the upregulation of intercellular communication, and the width of the line indicates the strength of up‐ or downregulation. (D) CellChat showing the differences in ligand–receptor pairs and molecular interactions of cell types between the two groups. (E) CellChat showing the overall signalling patterns in Scissor^low^ (left) and Scissor^high^ clusters (right). (F) Network plot showing the specific signalling pathways of intercellular communication between the two groups.

We applied the SHSS to the HNSCC scRNA‐seq samples and divided the samples into Scissor^high^ and Scissor^low^ groups (Figure 2A). CellChat was used to assess the cell interactions between the two groups. Intriguingly, we found that the cells in the Scissor^high^ group had a higher overall number and strength of interactions (Figure 2B). Specifically, endothelial cells in the Scissor^high^ group exhibited stronger interactions with tumour epithelial cells and weaker interactions among immune cell clusters (Figure 2C). Subsequently, we compared the differences in the ligand–receptor pairs and molecular interactions between the two groups. The results showed that cells in the Scissor^low^ group were enriched in immune activation pathway‐related molecules (*CD96*, *CD99* and *IL‐1*), consistent with the enhanced immune cell activity described above, whereas cells in the Scissor^high^ group were enriched in immunosuppressive pathway‐related molecules (*CD22* and *CD46*) as well as tumour‐associated pathway molecules (collagen and laminin) (Figure 2D–F). Overall, these results demonstrate the correlation of HNSCC TME activity with the SHSS group.

### 3.3 Gene mutation landscape of TCGA HNSCC samples based on SHSS classification

**FIGURE 3:**
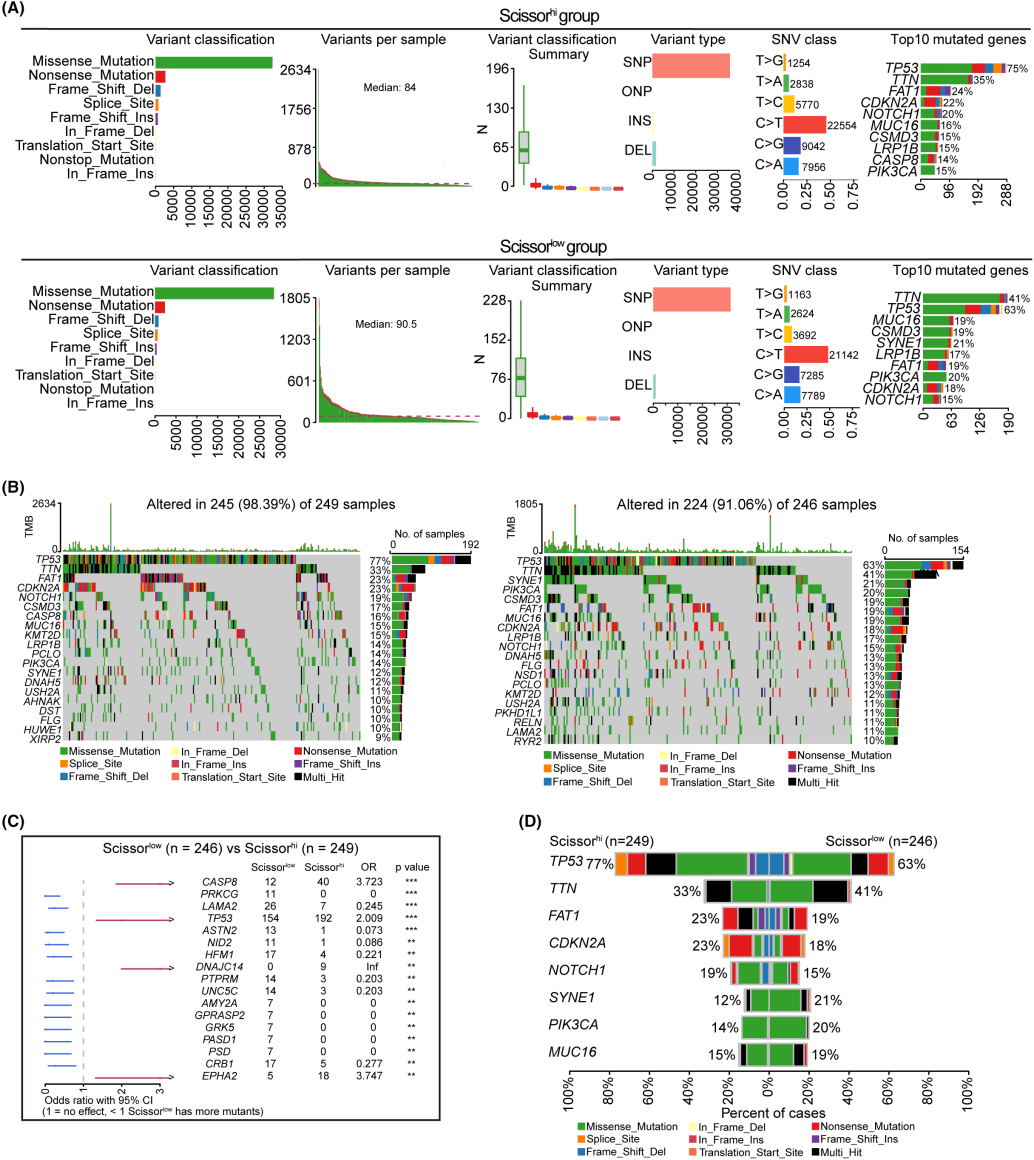
Gene mutation landscape in TCGA HNSCC samples in Scissor^low^ and Scissor^high^ clusters. (A) Variant classification, variant type, single‐nucleotide variant (SNV) class, mutation load per sample, summary of variant classification and top 10 mutated genes in the Scissor^high^ (left) and Scissor^low^ (right) groups are shown. (B) Oncoprint showing the most frequently mutated genes in the Scissor^high^ (upper) and Scissor^low^ (lower) groups. Sidebar graph showing the number of mutant genes. (C) Forest plots showing the top three differentially mutated genes between the two groups, and the bars indicate the odds of a 95% confidence interval. Neighbouring table includes the number of mutated HNSCC genes. (D) Barplot showing the most frequently mutated genes in the two groups.

Clinical therapy and patient prognosis are closely associated with mutation burden and frequency. Therefore, we linked the mutation data from TCGA HNSCC patients to the SHSS group. As shown in Figure 3A, the most common variant classification in both groups was missense mutation, the most common variant type was SNP and single‐nucleotide variant (SNV) CLASS was predominantly T > G. Unexpectedly, we found that the most of top‐10 mutated genes (*TP53*, *TTN*, *FAT1*, *CDKN2A*, *NOTCH1*, *MUC16*, *CSMD3*, *LRP1B* and *PIK3CA*) were the same and mutation frequency of them possessed unobvious difference in both groups (Figure 3A), which hinted that mutation frequency mentioned above nine genes play essential roles in HNSCC initiation and progression. Intriguingly, *CASP8* was one of the most frequently mutated gene only inthe Scissor^high^ group, while *SYNE1* only discovered in the Scissor^low^ group (Figure 3A), suggesting that mutation frequency of *CASP8* and *SYNE1* play an potential role to predict the prognosis in HNSCC. The total SNV frequency of genes in the Scissor^high^ group was 98.39% (245/249 samples) and the total SNV frequency of genes in the Scissor^low^ group was 91.06% (224/246 samples) (Figure 3B). Differentially mutated genes in the Scissor^high^ group were mainly *TP53*, *CASP8*, *DNAJC4* and *EPHA2*, whereas those in the Scissor^low^ group were mainly *PRKCG*, *LAMA2*, *HFM*, *PTPRM*, *UNC5C*, *AMY2A*, *GPRASP2*, *GRK5*, PASD1, PSD and *CRB1* (Figure 3C). In addition, the case percentage of high mutation frequency genes were different between the Scissor^high^ group and the Scissor^low^ group. Taken together, these data suggested that gene mutations are important causes and differentially mutated genes may be used to predict prognosis in HNSCC based on SHSS classification.

### 3.4 Scissor^high^ group exhibits low tumour immune cell infiltration

**FIGURE 4:**
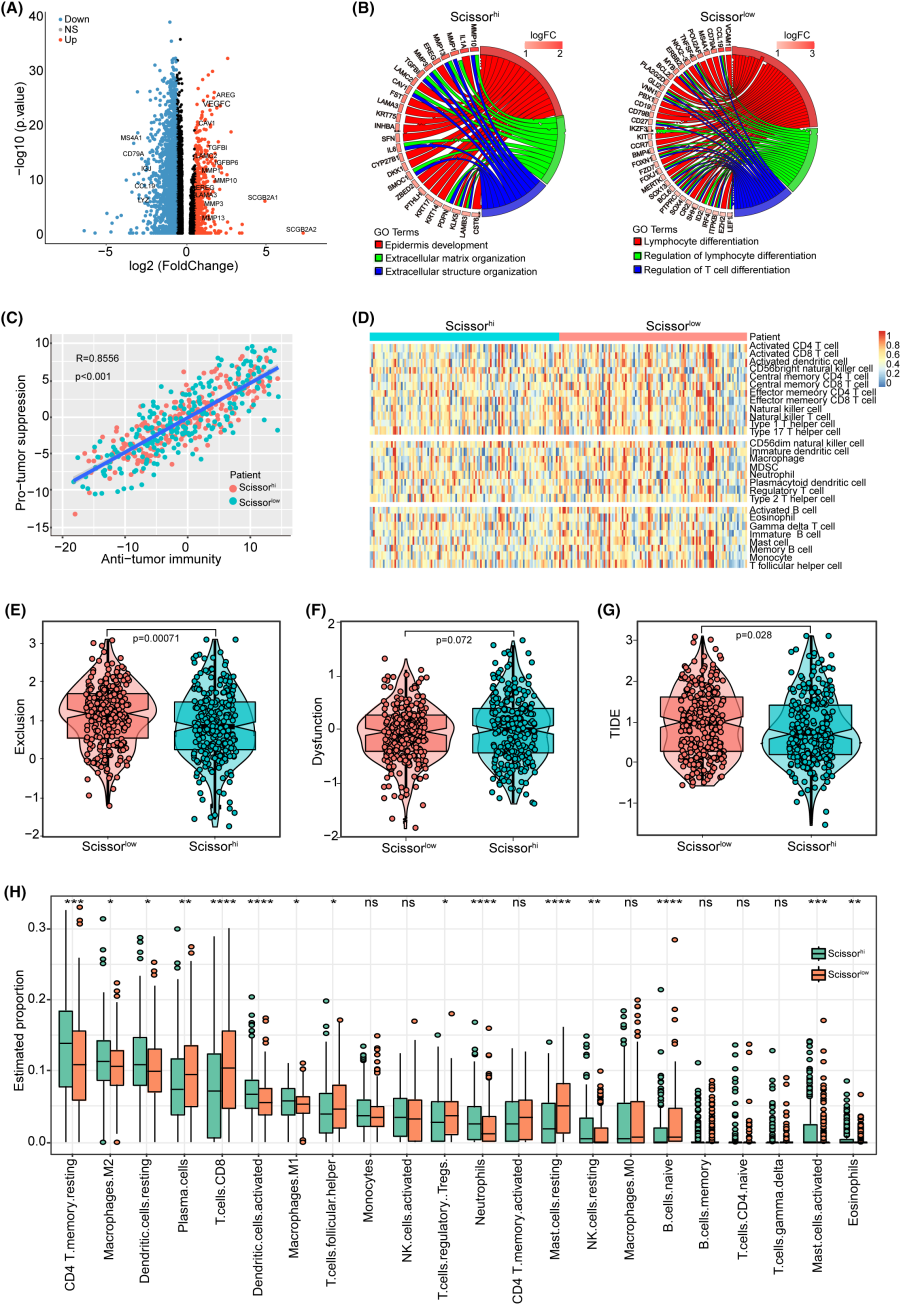
Molecular characteristics and tumour immune infiltration score of TCGA HNSCC dataset. (A) Volcano plot of differential gene expression between the two groups in TCGA HNSCC dataset. (B) Gene Ontology (GO) cluster plot showing a chord dendrogram of the clustering of the expression spectrum of significant genes in the Scissor^high^ (left) and Scissor^low^ (right) groups. (C) Correlation between pro‐tumour suppression and anti‐tumour immunity in TCGA HNSCC patient cohort. (D) Heatmap showing the enrichment levels of 28 immune‐related cells in the two groups via single‐sample gene set enrichment analysis (ssGSEA) analysis. (E–G) Violin plot showing the scores of exclusion (E), dysfunction (F), and tumour immune dysfunction and exclusion (TIDE) (G) between the two groups. (H) Barplot showing the enrichment levels of 22 immune‐related cells in the two groups via estimate analysis.

Next, we compared the transcriptome expression profiles of the Scissor^high^ and Scissor^low^ groups of TCGA_HNSCC samples. As shown in Figure 4A, compared to the Scissor^low^ group, 508 upregulated and 440 downregulated genes were identified in the Scissor^high^ group. Furthermore, GO enrichment analysis for DEGs was performed, and the Scissor^high^ group was mainly enriched in epidermal development, extracellular matrix organization, and extracellular structure organization pathways, whereas lymphocyte differentiation, regulation of lymphocyte differentiation and regulation of T‐cell differentiation pathways were enhanced in the Scissor^low^ group (Figure 4B).

We then analysed the relationship between tumour immune cell infiltration and grouping, and performed ssGSEA to assess the comparative abundance of 28 immune cells between the two groups. The results showed a positive ratio between anti‐tumor and pro‐tumour immunity in both groups of samples (*R* = 0.8556, *p* < 0.001) (Figure 4C), but the Scissor^low^ group had a higher abundance of immune infiltrates (Figure 4D), indicating that patients in the Scissor^low^ group had stronger antitumor immunity. In addition, we calculated the TIDE scores for both groups to assess the potential for tumour immune evasion. The results showed that the Scissor^low^ group had higher Exclusion, TIDE scores, and lower dysfunction scores than the Scissor^high^ group (Figure 4E–G), indicating a weaker immune evasion ability. We also assessed the infiltration scores of 22 stromal and immune cells in the tumour using the CIBERSORT function and observed higher infiltration of CD8^+^ T, plasma and activated dendritic cells and lower infiltration of CD4^+^ T memory cells, M2 macrophages and resting dendritic cells in the Scissor^low^ group (Figure 4H). Taken together, our analysis demonstrated that this method of patient grouping can effectively separate patients with different immune statuses and has the potential to guide immunotherapy.

### 3.5 Validation of the GEO_HNSCC patient cohort

**FIGURE 5:**
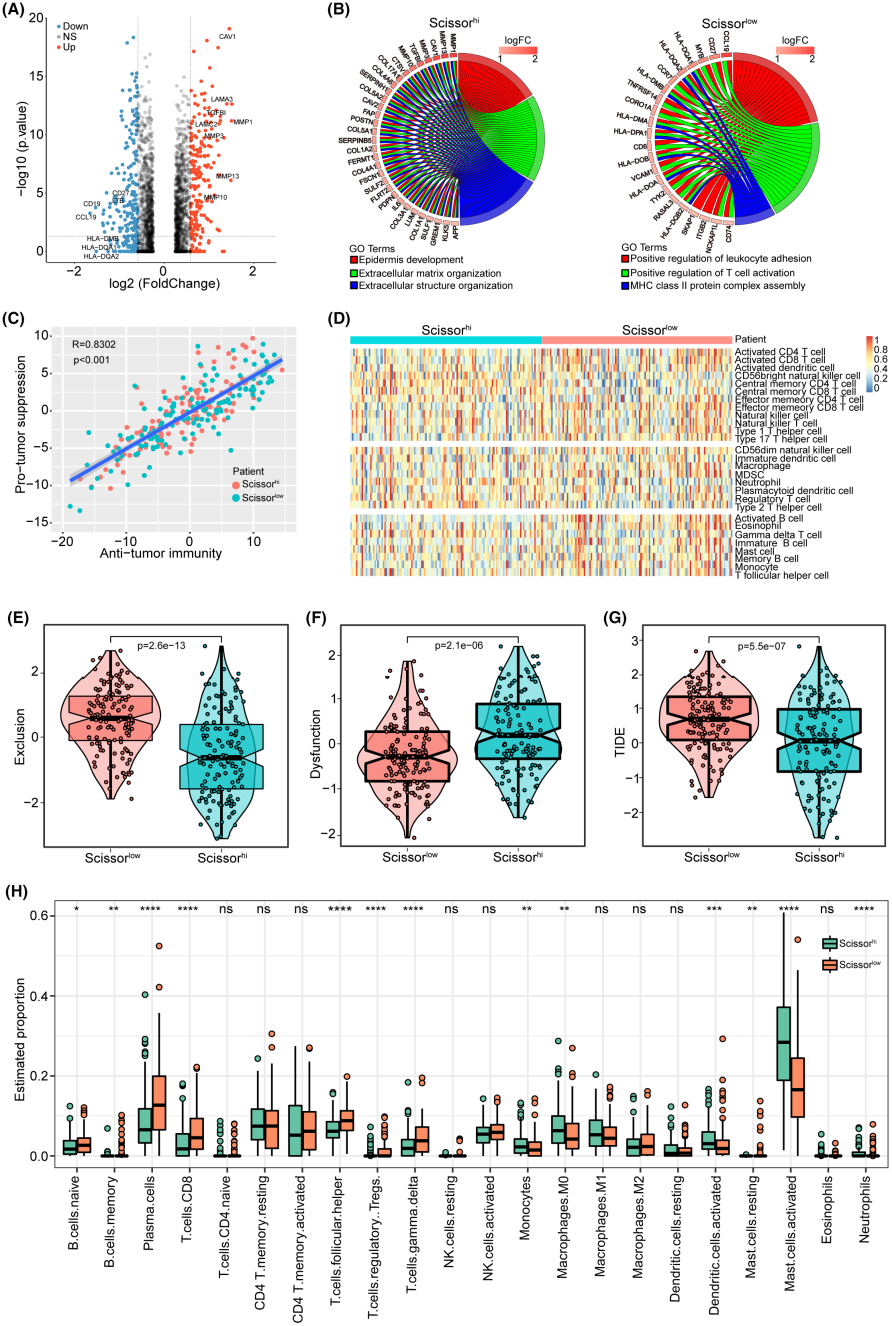
Molecular characteristics and tumour immune infiltration score of the GEO HNSCC dataset. (A) Volcano plot of differential gene expression between the two groups in the GEO HNSCC dataset. (B) GO cluster plot showing a chord dendrogram of the clustering of the expression spectrum of significant genes in the Scissor^high^ (left) and Scissor^low^ (right) groups. (C) Correlation between pro‐tumour suppression and anti‐tumour immunity in the GEO HNSCC patient cohort. (D) Heatmap showing enrichment levels of 28 immune‐related cells in the two groups via ssGSEA analysis. (E–G) Violin plot showing the scores of exclusion (E), dysfunction (F), and TIDE (G) between the two groups. (H) Barplot showing the enrichment levels of 22 immune‐related cells in the two groups via estimate analysis.

We further verified the consistency of the SHSS classification in HNSCC samples (GSE65858) from the GEO database. The volcano plot revealed 399 upregulated and 417 downregulated genes in the Scissor^high^ group (Figure 5A). GO functional enrichment results showed that the Scissor^high^ group was mainly involved in epidermal development, extracellular matrix organization and extracellular structure organization pathways, while the Scissor^low^ group was activated in the positive regulation of leukocyte cell–cell adhesion, positive regulation of T cell activation and MHC class II protein complex assembly pathway (Figure 5B). We also validated the relationship between the classification and tumour immune cell infiltration in the GEO cohort and showed a positive relationship between anti‐tumour and pro‐tumour immunity in both groups of samples (*R* = 0.8302, *p* < 0.001) (Figure 5C) and a higher abundance of immune infiltration in the Scissor^low^ group (Figure 5D), which is consistent with TCGA results. In addition, we validated the TIDE scores in both groups to assess the possibility of tumour immune evasion. The results showed that the Scissors^low^ group had higher Exclusion, TIDE scores and lower dysfunction scores than the Scissors^high^ group (Figure 5E–G), indicating weaker immune evasion. CIBERSORT function showed higher infiltration of CD8^+^ T and plasma cells and lower infiltration of activated mast cells in the Scissor^low^ group (Figure 5H). Taken together, our data demonstrate the consistency of this classification in the TCGA and GEO cohorts.

### 3.6 SHSS model for predicting the survival of patients with HNSCC

**FIGURE 6:**
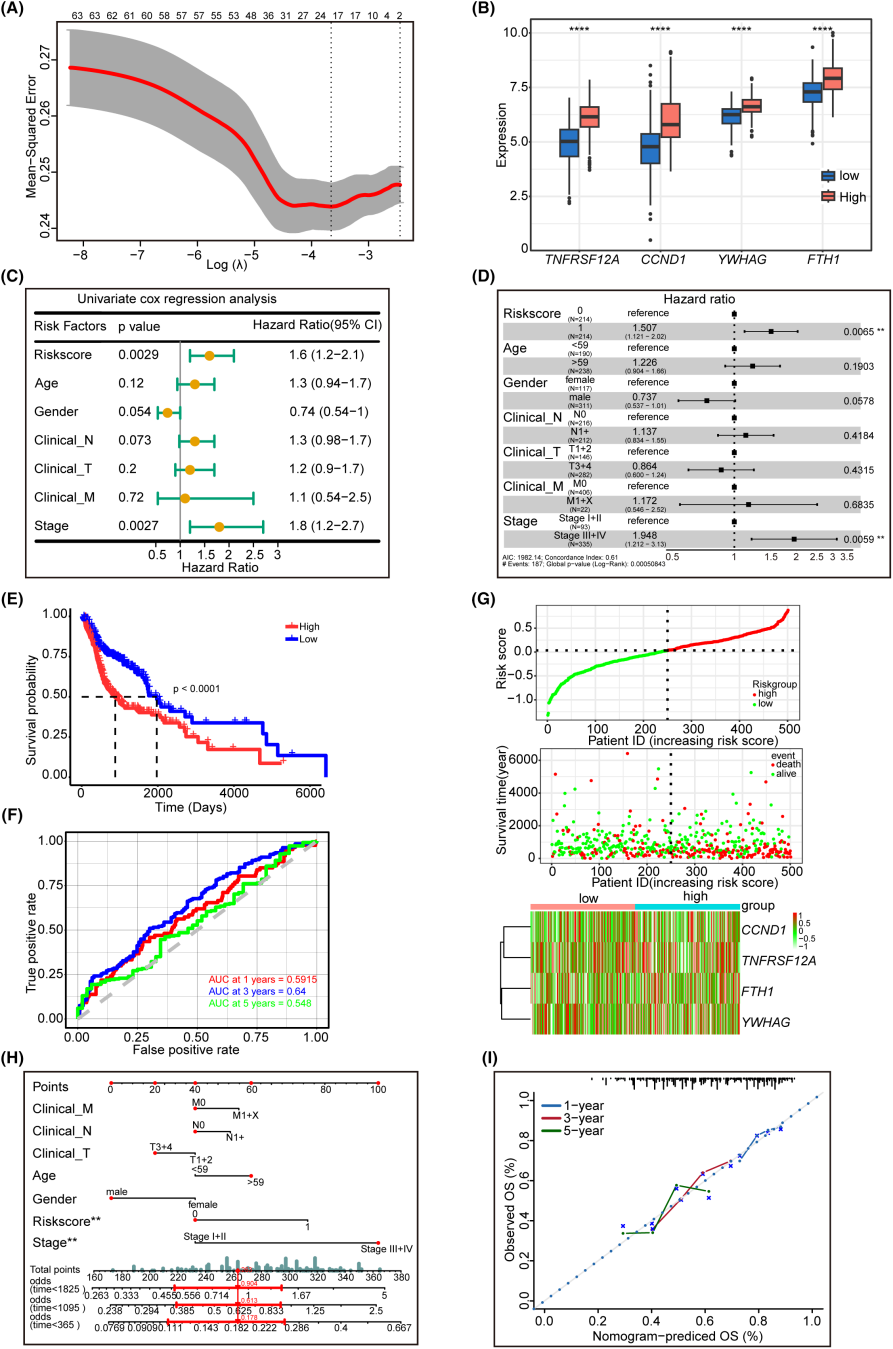
Construction of the prognostic risk model using the Scissor^+^ endothelial characteristic genes in HNSCC TCGA datasets. (A) LASSO coefficient profiles: A coefficient profile plot was plotted against the log (λ) sequence. (B) Boxplot showing the expression levels of modelling‐related genes in the two groups. (C) Forest map showing overall survival (OS)‐related clinical factors using single‐factor Cox regression analysis. (D) Forest map showing the parameters associated with survival using multiple regression logistic analysis. (E) Kaplan–Meier survival curves established using prognosis‐related risk scores. (F) Receiver operating characteristic (ROC) curve graphs for OS at 1, 3, and 5 years. (G) The model classified the patients in the training set into low‐ and high‐risk groups. (H and I) Nomogram models (H) and calibration curves (I) to predict the OS in patients with HNSCC at 1, 3, and 5 years.

**FIGURE 7:**
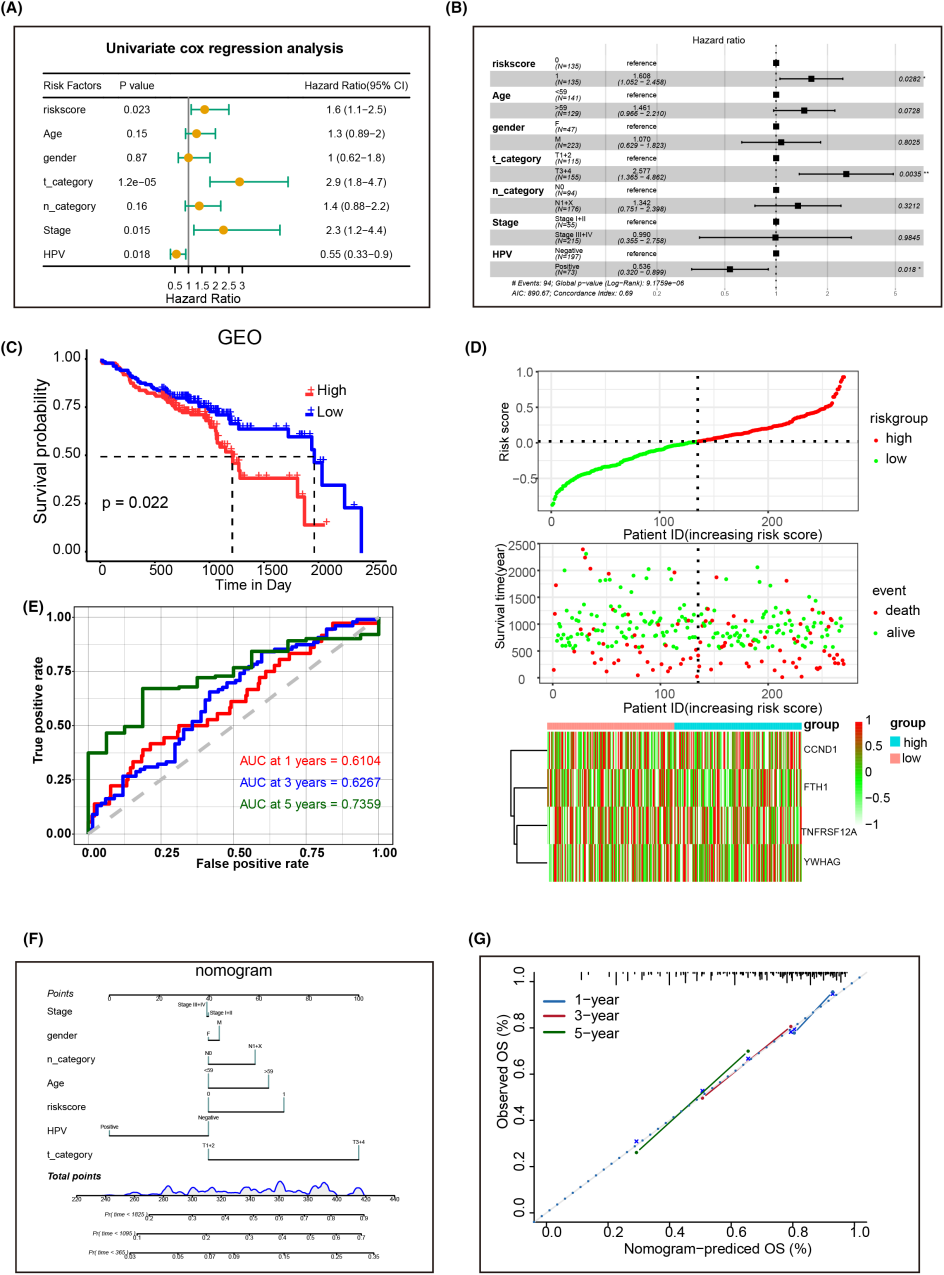
Validation of the prognostic risk model for HNSCC using GEO datasets. (A and B) Forest map showing OS‐related clinical factors using single‐factor (A) and multi‐factor (B) Cox regression analyses. (C) Kaplan–Meier survival curves established using prognosis‐related risk scores. (D) The model classified the patients in the training set into low‐ and high‐risk groups. (E) ROC curve graphs for OS at 1, 2, and 3 years. (F and G) Nomogram models (F) and calibration curves (G) to predict the OS of patients with HNSCC at 1, 2, and 3 years.

We conducted LASSO regression analysis to optimize the SHSS to build a prognostic risk model and identified four genes, *TNFRSF12A*, *CCND1*, *YWHAG* and *FTH1* (Figure 6A), which were all expressed higher in the high‐risk group (Figure 6B). Based on their coefficients, we calculated the risk score using the following formula: risk score = expression level of *CCND1* × 0.100+ expression level of *FTH1* × 0.212+ expression level of *TNFRSF12A* × (0.177) + expression level of *YWHAG* × (0.260). We used univariate and multivariate Cox regression analyses on TCGA and GSE65858 cohorts to examine whether the risk score could predict prognosis independently of traditional clinical factors. The results demonstrated that the risk score (HR: 1.6, 1.507; 95% CI: 1.2–2.1 and 1.121–2.02, respectively) was an independent predictor of OS (Figure 6C,D). Similarly, risk scores (HR: 1.6 and 1.608, respectively; 95% CI: 1.1–2.5 and 1.052–2.458) obtained in the GSE65858 cohort were independent predictors of OS (Figure 7A,B). Based on the median risk score of TCGA and GSE65858 cohorts, patients were divided into high‐risk and low‐risk groups. KM and log‐rank analyses revealed that in both cohorts, HNSCC patients in the high‐risk group had worse OS than those in the low‐risk group (Figure 6E and Figure 7C). The overall risk score (top panel), survival time (middle panel) and gene expression levels (bottom panel) for TCGA and GSE65858 datasets are shown in Figure 6F and Figure 7D. Furthermore, risk scores functioned effectively in predicting OS in the TCGA (AUC for 1‐, 3‐ and 5‐year OS: 0.5915, 0.64 and 0.548, respectively; Figure 6G) and GSE65858 cohorts (AUC for 1‐, 2‐ and 3‐year OS: 0.6104, 0.6267, and 0.7359, respectively; Figure 7E). Nomograms with different components to predict 1‐, 3‐ and 5‐year OS for TCGA dataset (Figure 6H) and 1‐, 2‐ and 3‐year OS for GSE65858 dataset were generated (Figure 7F). Calibration curves revealed that the nomograms were adequately calibrated across all the datasets (Figure 6I and Figure 7G).

### 3.7 Validation of the prognostic model via the clinical cohort

**FIGURE 8:**
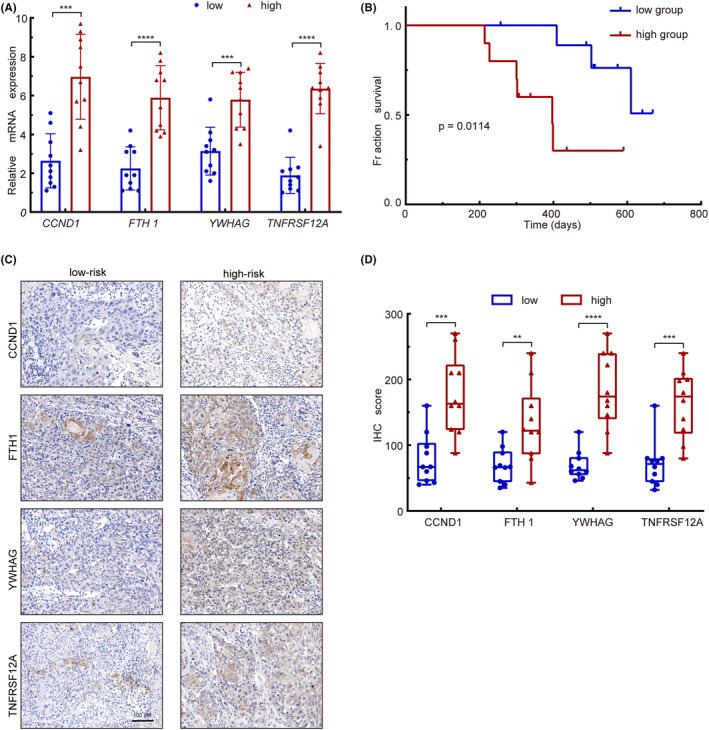
Validating the risk model genes in a clinical group. (A) Expression levels of the four genes were compared between the high‐risk and low‐risk groups. (B) Kaplan–Meier survival curves of HNSCC patients based on four genes Risk‐scores. (C and D) Overexpression of risk model genes was observed in the high‐risk group of patients. Scale bar, 100 μm. *p < 0.05, **p < 0.01, ***p < 0.001, ****p < 0.0001.

To substantiate the prognostic significance of our model, we recruited a clinical cohort consisting of 20 patients with head and neck squamous cell carcinoma at various clinical stages to verify the expression of the four genes (*CCND1*, *FTH1*, *YWHAG* and *TNFRSF12A*). By directly quantifying mRNA levels of *CCND1*, *FTH1*, *YWHAG* and *TNFRSF12A*, we aimed to reinforce the association between these genes and our risk model. RT‐qPCR's sensitivity and specificity allowed us to precisely distinguish between low‐ and high‐risk patients within our clinical cohort according to the expression levels of the risk model four genes. The mRNA levels of four genes were obtained by RT‐qPCR for each patient as shown in Table S1. We found that these four genes were significantly high expression in the high‐risk group (Figure 8A). Kaplan–Meier analysis showed that higher expressions of the risky four genes were significantly associated with poorer survival of HNSCC patients (*p* = 0.0114) (Figure 8B). Complementing RT‐qPCR, IHC analysis brought spatial and protein‐level insights into our validation process. Immunohistochemistry (IHC) analysis also substantiated that four genes were overexpressed in high‐risk patients (Figure 8C,D), further strengthening the link between these genes and adverse patient outcomes. All above experimental results were consistent with our constructed risk model, indicating that these four genes serve as potential prognosis biomarkers for predicting the HNSCC patients' survival.

## 4 DISCUSSION

Malignant tumour heterogeneity has been a major focus of research in recent decades,^17^, ^18^ offering insights into novel tumour subtypes and potential therapeutic avenues. The identification of novel tumour subtypes, particularly the discovery of new cell clusters and markers, is an effective approach to unravel tumour heterogeneity and provide more personalized therapeutic options for clinical use. Single‐cell transcriptomic technology has proven to be an effective method for discovering novel cell subpopulations and biomarkers in malignant tumours.^21^, ^22^

In this study, we identified five cell clusters using online HNSCC scRNA‐seq data from the GEO database: fibroblasts, epithelial cells, endothelial cells, T cells, myeloid cells and B cells. TME is composed of malignant cells, immune cells, endothelial cells and fibroblasts, all of which play crucial roles in regulating various aspects of tumour behaviour, including proliferation, metastasis and drug resistance.^1^, ^2^, ^3^ Malignant cells can self‐secrete immunosuppressive substances, such as interleukin‐2, interleukin‐10, transforming growth factor and vascular endothelial growth factor, into the TME to modulate immune cells and inhibit their anti‐tumour activity.^23^ In contrast, endothelial cells can also exert pro‐tumour activity; for example, vascular endothelial cells can regulate *SOX7* expression through the *ASK1‐c‐Jun* signalling pathway, thereby promoting angiogenesis and tumour growth.^24^ Another study revealed that tumour‐associated endothelial cells could activate platelets and increase tumour vascular endothelial permeability by inhibiting VE‐calmodulin to promote tumour metastasis.^25^

Database‐based bioinformatic analysis has demonstrated its superiority and clinical applications.^26^, ^27^ To pinpoint subpopulations of endothelial cells with prognostic significance, we employed the Scissor function. The resulting SHSS was characterized by specific gene expression patterns, which were instrumental in stratifying HNSCC patients from both TCGA and GEO cohorts based on overall survival (OS) rates. We observed that patients in the Scissorhigh group had worse OS rates, and cells in this group tended to exhibit increased interactions between immune cells and tumour epithelial cells, along with decreased interactions between immune cells.

Identifying genomic drivers in different subpopulations is crucial. CNV analysis showcased that while top mutated genes were shared between Scissor^low^ and Scissor^high^ groups, their mutation frequencies varied, indicating their pivotal roles in HNSCC development. Notably, the mutation frequency of *CASP8*, closely related to tumour risk,^28^ was elevated in the Scissor^high^ group, while mutated *SYNE1* was mainly occurred in the Scissor^low^ group. Moreover, GO enrichment analysis revealed that patients in the Scissor^low^ group were primarily enriched in pathways related to immune cell activation, which also demonstrated previous activation of immune cells in the Scissor^low^ group. Therefore, the SHSS is an effective method for HNSCC classification.

Tumour immune cell infiltration profoundly impacts patient outcomes. In this study, we investigated and validated the effect of SHSS classification on immune cell infiltration based on bulk RNA‐Seq data. The ssGSEA, TIDE and CIBERSORT results demonstrated a higher abundance of immune infiltration and higher immune activity in the Scissor^low^ group. Based on these findings, we propose that endothelial cells with higher SHSS scores may play a pivotal role in establishing or reinforcing an immunosuppressive TME. This interpretation aligns with the notion that certain endothelial cell subpopulations may contribute to the establishment of an environment that dampens immune surveillance, promoting tumour progression and evasion from immune attacks.^30^ It is essential to acknowledge that the interaction between endothelial cells and the immune system is intricate and multifaceted, and further in‐depth investigations are warranted to dissect the underlying molecular mechanisms governing these interactions. In conclusion, endothelial cells with higher SHSS scores in patients may play a key role in shaping or enhancing the immunosuppressive TME.

Previous studies have demonstrated that gene signatures can be effective in cancers to guide treatment or determine prognosis, such as the 21‐gene panel for assessing breast cancer recurrence^29^ and the 18‐gene signature for assessing recurrence risk in patients with stage II colon cancer.^30^ We identified four candidate genes using LASSO and random forest survival models that could predict overall patient survival effectively with high AUC values. Specifically, elevated *TNFRSF12A* expression consistently correlates with increased cellular proliferation, migration, and invasiveness.^31^, ^32^ In diverse malignancies, it orchestrates tumour growth and metastasis, aligning with the aggressive behaviour in HNSCC. Dysregulation of *CCND1* is intricately interwoven with HNSCC, driving uncontrolled cellular proliferation—a hallmark of cancer.^33^, ^34^ *YWHAG* expression perturbations align with intricate molecular interactions dictating tumour behaviour and therapy response.^35^, ^36^, ^37^ *YWHAG* may extend to HNSCC pathogenesis, activating tumorigenic pathways and influencing disease progression. *FTH1* plays an essential role in intracellular iron storage, impacting survival, proliferation and drug resistance dynamics.^38^, ^39^ These genes unveil a complex molecular landscape potentially influencing HNSCC progression and offering potential therapeutic insights. Our predictive model, incorporating these biomarkers, can stratify patients by risk, guiding tailored treatments. High‐risk patients may access intensive monitoring, novel interventions, or clinical trials, while low‐risk patients could avoid unnecessary aggressive treatments. Integrating our model with routine assessments offers a comprehensive prognosis view, assisting informed decisions and optimizing treatments for improved outcomes. Our study's pathway for validation and implementation in larger cohorts underscores its clinical value. By bridging the gap between research and practical applications, we emphasize the potential of our prognostic prediction model to enhance cancer prognosis and benefit patients.

In this study, we analysed the gene expression profiles of patients with HNSCC from the scRNA‐seq and bulk RNA‐Seq data, and validated the prognostic model by RT‐qPCR and IHC analysis of 20 patients. We found that SHSS is associated with the OS of patients with HNSCC by analysing the malignant endothelial cells. Furthermore, experimental results sustained that the risk model could predict the prognosis and immune response in HNSCC. In summary, these results improve our understanding of the heterogeneity of the TME at the single‐cell level and provide a 4‐gene prognostic model for HNSCC.

## AUTHOR CONTRIBUTIONS

**Chunlong Yang:** Conceptualization (equal); funding acquisition (equal); validation (lead); writing – original draft (equal). **Xiaoning Cheng:** Data curation (equal); validation (supporting); writing – review and editing (equal). **Shenglan Gao:** Data curation (equal); validation (supporting); writing – review and editing (equal). **Qingjun Pan:** Conceptualization (equal); methodology (equal); validation (equal); writing – review and editing (equal).

## FUNDING INFORMATION

This work is supported by Natural Science Foundation of Guangdong (2022A1515111006, 2021A1515010827).

## CONFLICT OF INTEREST STATEMENT

The authors declare that they have no known competing financial interests or personal relationships that could have appeared to influence the work reported in this paper.

## Supporting information


Table S1:

## Data Availability

The data that support the findings of this study are openly available in TCGA (https://www.cancer.gov/tcga) and GEO (http://www.ncbi.nlm.nih.gov/geo/) databases. The original data of Figure 8 are available in the Table S1 of this article.
